# Characterization of unique functionalities in c-Src domains required for osteoclast podosome belt formation

**DOI:** 10.1016/j.jbc.2021.100790

**Published:** 2021-05-18

**Authors:** Takuma Matsubara, William N. Addison, Shoichiro Kokabu, Lynn Neff, William Horne, Francesca Gori, Roland Baron

**Affiliations:** 1 Division of Bone and Mineral Research, Oral Medicine, Infection and Immunity, Harvard School of Dental Medicine, Boston, Massachusetts, USA; 2 Division of Molecular Signaling and Biochemistry, Department of Health Improvement, Kyushu Dental University, Fukuoka, Japan; 3 Department of Medicine, Harvard Medical School and Endocrine Unit, MGH, Boston, Massachusetts, USA

**Keywords:** osteoclast, Src, Src homology 3 domain (SH3 domain), Src homology 2 domain (SH2 domain), podosome, CSK, C-terminal Src kinase, MOI, multiplicity of infection, PBS, phosphate buffered saline, PPR, proximal proline-rich, qPCR, real-time quantitative PCR, RANKL, receptor activator of nuclear factor kappa-B ligand, SFK, Src family kinase, TRAP, tartrate-resistant acid phosphatase

## Abstract

Deletion of c-Src, a ubiquitously expressed tyrosine kinase, results in osteoclast dysfunction and osteopetrosis, in which bones harden into “stone.” In contrast, deletion of the genes encoding other members of the Src family kinase (SFK) fails to produce an osteopetrotic phenotype. This suggests that c-Src performs a unique function in the osteoclast that cannot be compensated for by other SFKs. We aimed to identify the molecular basis of this unique role in osteoclasts and bone resorption. We found that c-Src, Lyn, and Fyn were the most highly expressed SFKs in WT osteoclasts, whereas Hck, Lck, Blk, and Fgr displayed low levels of expression. Formation of the podosome belt, clusters of unique actin assemblies, was disrupted in *src*^*−/−*^ osteoclasts; introduction of constitutively activated SFKs revealed that only c-Src and Fyn could restore this process. To identify the key structural domains responsible, we constructed chimeric Src–Hck and Src–Lyn constructs in which the unique, SH3, SH2, or catalytic domains had been swapped. We found that the Src unique, SH3, and kinase domains were each crucial to establish Src functionality. The SH2 domain could however be substituted with Lyn or Hck SH2 domains. Furthermore, we demonstrate that c-Src’s functionality is, in part, derived from an SH3–proximal proline-rich domain interaction with c-Cbl, leading to phosphorylation of c-Cbl Tyr700. These data help clarify Src’s unique functionality in the organization of the cytoskeleton in osteoclasts, required for efficient bone resorption and explain why c-Src cannot be replaced, in osteoclasts, by other SFKs.

The ubiquitously expressed rous sarcoma oncogene tyrosine kinase (Src) is important in cell proliferation, adhesion, and migration (1). Despite its widespread expression in multiple cell types, the major phenotype of *src*^−/−^ mice is osteopetrosis caused by defective osteoclast bone resorption (2), suggesting that Src fulfills a unique, nonredundant function in osteoclasts. Among the Src family kinases (SFKs), only the deletion of Src results in osteopetrosis (3, 4, 5). It has been shown that the mechanism by which Src deletion affects bone resorption is for the most part by altering a signaling pathway that governs the formation, turnover, and organization of podosomes in a peripheral belt in active osteoclasts attached to the bone (6). Some degree of redundancy may exist however within the SFK family of tyrosine kinases because *src*^*−/−*^;*hck*^*−/−*^ double-KO mice are more osteopetrotic than Src-deficient mice (7). In contrast, deleting Lyn increases receptor activator of nuclear factor kappa-B ligand (RANKL)-induced osteoclastogenesis, resulting in osteopenia rather than osteopetrosis, and has little effect on the activity of mature osteoclasts (8, 9). These reports suggest that while some other SFKs compensate for the absence of Src in most tissues and Hck may partially compensate for Src’s absence in osteoclasts, Src is specifically and uniquely required for the function of mature osteoclasts, more specifically the organization of their cytoskeleton.

Src plays a central role in integrin-mediated cell adhesion and the formation of podosomes, the specialized adhesion structures that are required for normal osteoclastic bone resorption (10). Src is activated downstream of α_v_β_3_ integrin, which mediates the attachment of osteoclasts to bone matrix and contributes to the migration of osteoclasts over the bone surface and the formation of the sealing zone that surrounds and delineates the extracellular bone resorbing compartment (10, 11, 12, 13). In this area, Src regulates the formation and organization of podosomes, the dynamic punctate actin adhesion structures that are found in osteoclasts and other highly motile cells (11, 14). Consequently, Src-deficient osteoclasts are not only unable to resorb the bone but also 50 to 60% less mobile than WT osteoclasts (15, 16). Our studies also indicate that Src promotes the initiation of podosome formation, the rate of actin polymerization within podosomes, the disassembly of podosomes, and the organization of the peripheral podosome belt by phosphorylating some of the many proteins that are known to be Src substrate proteins and to regulate podosome function (14, 17).

All members of the SFK family have similar domain structures with four distinct domains: unique, Src homology (SH)3, SH2, and kinase (catalytic) domains (18, 19, 20). Unique domains contain myristoylation and palmitoylation sites that mediate membrane association, but otherwise, there is little homology between any of the SFK unique domains (21). In contrast, SH3 and SH2 domains contain conserved and specific amino acid sequences that bind to proximal proline-rich (PPR) motifs and sequences containing phosphotyrosines, respectively, on other signaling proteins to form signaling complexes. The SFK SH3 and SH2 domains also bind intramolecularly to downregulate Src/SFK kinase activity (20, 22, 23, 24). The kinase domain phosphorylates components of the signaling complexes on tyrosine residues (25).

To delineate the molecular basis for the specific role of Src in osteoclasts, we first determined the relative expression level of other Src family kinases in these cells and analyzed the effects of expressing these in *src*^−/−^ osteoclasts. We found that Lyn is upregulated in *src*^−/−^ osteoclasts but neither compensates for Src’s absence nor represses Src’s ability to promote cell spreading and podosome belt formation. To identify the features of Src that are the basis of the kinase’s unique role in regulating osteoclast function, we expressed chimeric Hck–Src and Lyn–Src proteins in an open conformation and determined how replacing individual Src domains by the homolog domain from these other SFKs altered osteoclast spreading and podosome belt formation. The results indicate that Src’s unique, SH3, and kinase, but not the SH2, domains are required for the specific Src functions in osteoclasts.

## Results

### Lyn is highly expressed in src^−/−^ osteoclasts but does not contribute to the src^−/−^ osteoclast phenotype

**Figure 1:**
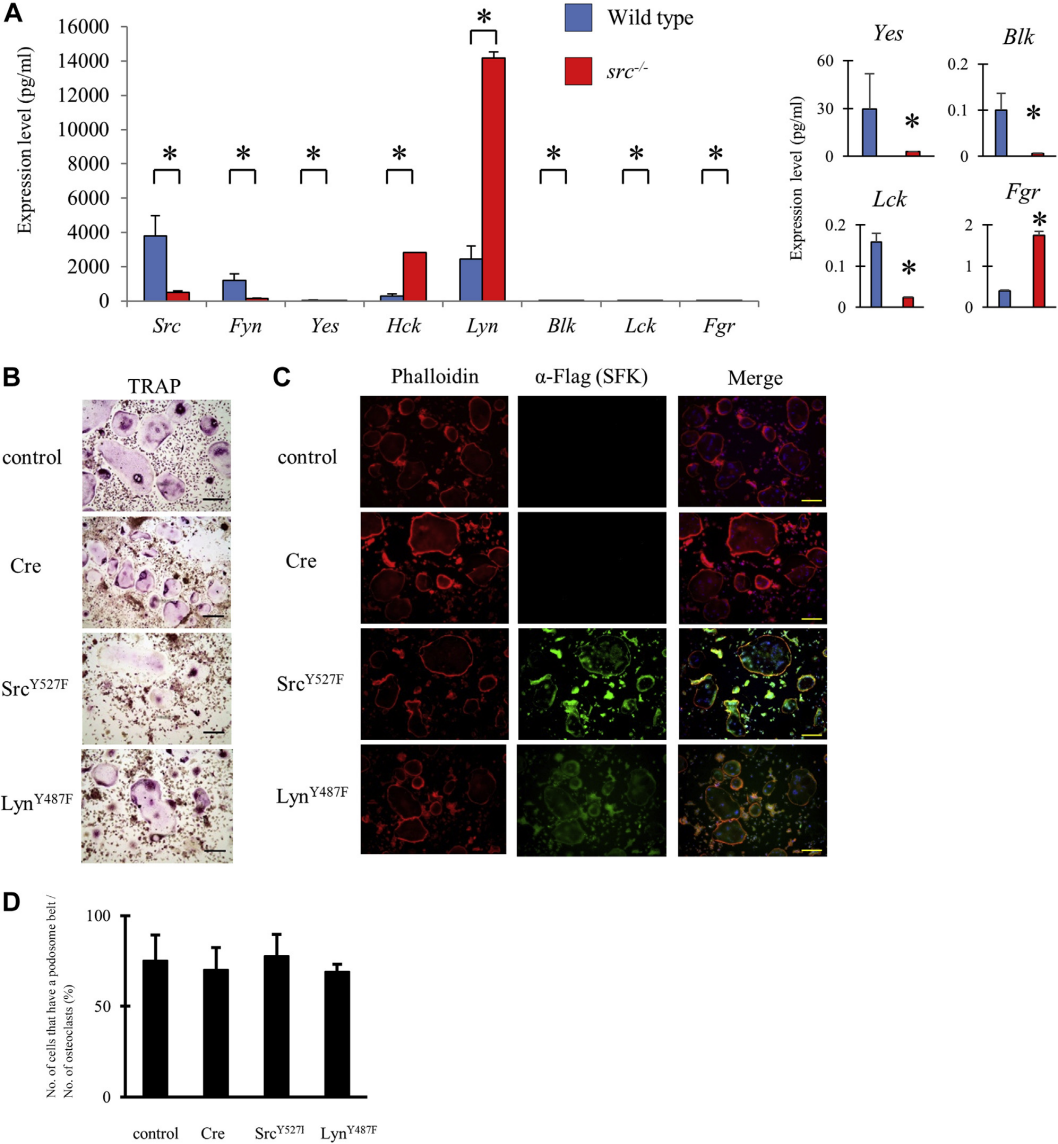
**Lyn expression was greatly increased in *src***^**−/−**^**tartrate-resistant acid phosphatase–positive multinuclear cells (osteoclasts) but increased expression of Lyn did not contribute to the *src***^**−/−**^**osteoclasts actin disorganization.***A*, spleen cells from WT (shown as WT) or *src*^−/−^ mice were cultured with M-CSF (30 ng/ml) and sRANKL (100 ng/ml). mRNA was isolated from osteoclasts, and the expression level was measured by real-time PCR. cDNAs of Src family kinases on the pcDNA3.1 vector were used to generate standard curves. A magnified graph (*Yes*, *Blk*, *Lck*, and *Fgr*) is shown at the *right side*. (Mean ± SD; n = 3). ∗*p* < 0.01 *versus* WT. *B* and *C*, WT spleen cells were differentiated to multinuclear cells and then infected with Cre adenovirus (MOI 50) without or with constitutively active Src or Lyn adenovirus (MOI 50). Multinuclear cells were stained by TRAP (*B*) or immunostained by rhodamine phalloidin (*red*), anti-Flag/anti-mouse Alexa Fluor 488 (*green*) and 4′, 6-diamidino-2-phenylindole (DAPI, *blue*) (*C*) 1 day after infection. The scale bar indicates 100 μm. *D*, osteoclasts and the cells having podosome belt were counted to obtain the ratio. (Mean ± SD; n = 4). There are no significant differences between all group. M-CSF, macrophage colony-stimulating factor; MOI, multiplicity of infection; sRANKL, soluble receptor activator of nuclear factor kappa-B ligand; TRAP, tartrate-resistant acid phosphatase.

To determine whether Src’s unique functional role in osteoclasts is only due to the particularly high Src level of expression relative to other SFKs, we first measured the expression level of all SFKs in WT osteoclasts by real-time quantitative PCR (qPCR) (Fig. 1*A*). Src and Lyn were more highly expressed than the other SFKs, with Lyn expression about 75% of Src expression. Fyn expression was about one third of Src expression and the expression of all the other SFKs was less than 10% of the Src level. Thus, Src, Lyn, and Fyn are the major SFKs in osteoclasts.

**Figure 2:**
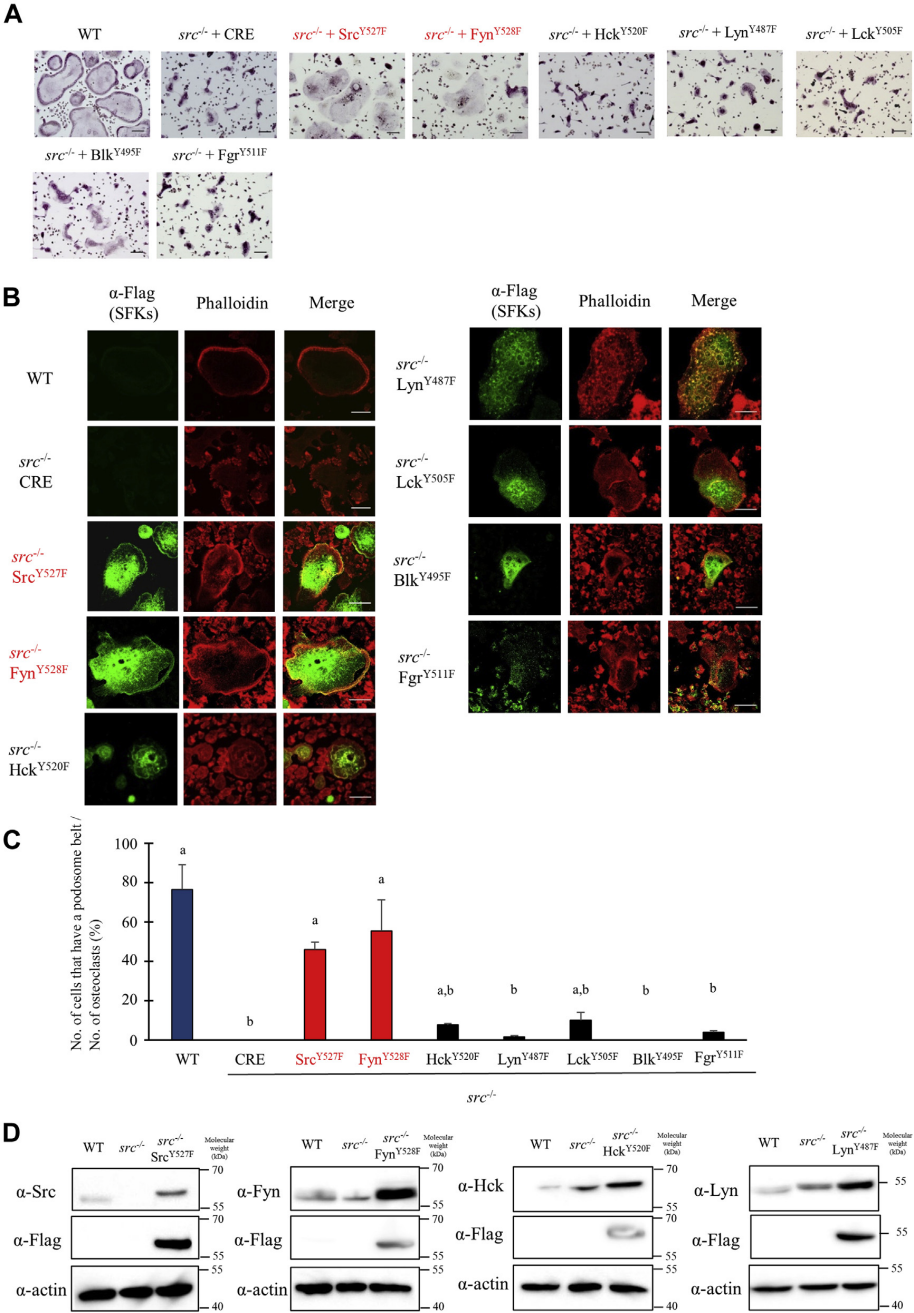
**Only active Src and Fyn rescued the *src***^***−/−***^**osteoclast phenotype.***A*–*C*, WT or *src*^*−/−*^ spleen cells were differentiated to tartrate-resistant acid phosphatase–positive multinuclear cells (osteoclasts) with sRANKL and M-CSF stimulation for 5 days. The cells were infected with Cre adenovirus (MOI 50) and SFK adenoviruses (MOI 50) as indicated and cultured with sRANKL and M-CSF. After a day, culture with adenovirus cells was fixed and stained by TRAP (*A*) or immunostained by rhodamine phalloidin (*red*), anti-Flag/anti-mouse Alexa Fluor 488 (*green*) and DAPI (*blue*) (*B*). Osteoclasts and the cells having podosome belt were counted to obtain the ratio. (Mean ± SD; n = 4). “a” denotes *p* < 0.01 *versus src*^*−/−*^ with Cre. “b” denotes *p* < 0.01 *versus* WT (*C*). *src^-/-^* osteoclasts were infected cre and indicated SFKs adenovirus. Expression level of indicated SFKs was determined by western blotting analysis (*D*). M-CSF, macrophage colony-stimulating factor; SFKs, Src family kinases; sRANKL, soluble receptor activator of nuclear factor kappa-B ligand; TRAP, tartrate-resistant acid phosphatase.

To determine whether Src deletion affects the expression level of other SFKs, possibly attempting to compensate the absence of Src, we next measured SFK expression in *src*^−/−^ osteoclasts. As shown in Figure 1*A*, in *src*^−/−^ osteoclasts, Lyn, Hck, and Fgr were significantly upregulated. Lyn expression increased to almost 4-fold higher than the expression of Src in WT osteoclasts, and the expression of Hck increased by 10-fold, reaching the level of Lyn expression in WT osteoclasts. In contrast, expression of Fyn was significantly lower in the *src*−/− osteoclasts and expression of the other SFKs remained very low (Fig. 1*A*). Corresponding with mRNA data, protein levels of Hck and Lyn in *src*^*−/−*^ osteoclasts were about 4- to 5-fold higher than that of WT osteoclasts (Fig. 2*D*, Fig. S1*A*). Fyn in *src*^*−/−*^ osteoclasts was about 60 to 70% of that in WT osteoclasts (Fig. 2*D*, Fig. S1*A*).

It has been reported that overexpression of Lyn suppresses osteoclast differentiation (8). Thus, it is possible that high levels of Lyn in *src*^−/−^ osteoclasts can suppress mature osteoclast function. To determine whether high levels of Lyn have a negative effect on podosome belt formation, we overexpressed constitutively active Lyn^Y487F^ in mature WT osteoclasts using an adenovirus system. Mature WT osteoclasts were large and multinuclear and formed podosome belts (Fig. 1, *B*–*D*). Infection with the CRE, Src^Y527F^, and/or Lyn^Y487F^ adenoviruses had little effect on tartrate-resistant acid phosphatase (TRAP) staining and podosome belt formation of the WT osteoclasts. These results suggest that high levels of Lyn are not likely to lead to a disruption in actin organization.

### Fyn compensates partially for c-Src function in src^−/−^ osteoclasts, but other Src family kinases do not

To determine which SFKs can support normal actin organization in osteoclasts, we introduced constitutively active SFKs into mature *src*^−/−^ osteoclasts. Mature WT osteoclasts differentiated from spleen cells with macrophage colony-stimulating factor and RANKL spread and had a round shape (Fig. 2*A*). F-actin in WT osteoclasts was organized in peripheral podosome belts (Fig. 2, *B* and *C*). In contrast, *src*^−/−^ osteoclasts were not well spread, had irregular forms (Fig. 2*A*), and failed to form podosome belts (Fig. 2, *B* and *C*), as previously described (14). As expected, infection of *src*^−/−^ osteoclasts with CRE adenovirus did not affect cell shape or actin organization. Introduction of the constitutively active Src^Y527F^ rescued the cell spreading and podosome belt formation of *src*^−/−^ osteoclasts as previously reported (14, 17) (Fig. 2). Interestingly, Fyn^Y528F^ also restored effectively the cell spreading, shape, and podosome belt formation of *src*^*−/−*^ osteoclasts, suggesting that the failure of Fyn to compensate for the absence of Src was the consequence of low expression level (Fig. 2*A*). On the other hand, induction of other SFKs did not rescue podosome belt formation of *src*^−/−^ osteoclasts. (Fig. 2, *B*–*D*).

### Src’s catalytic domain confers functional specificity in osteoclasts

**Figure 3:**
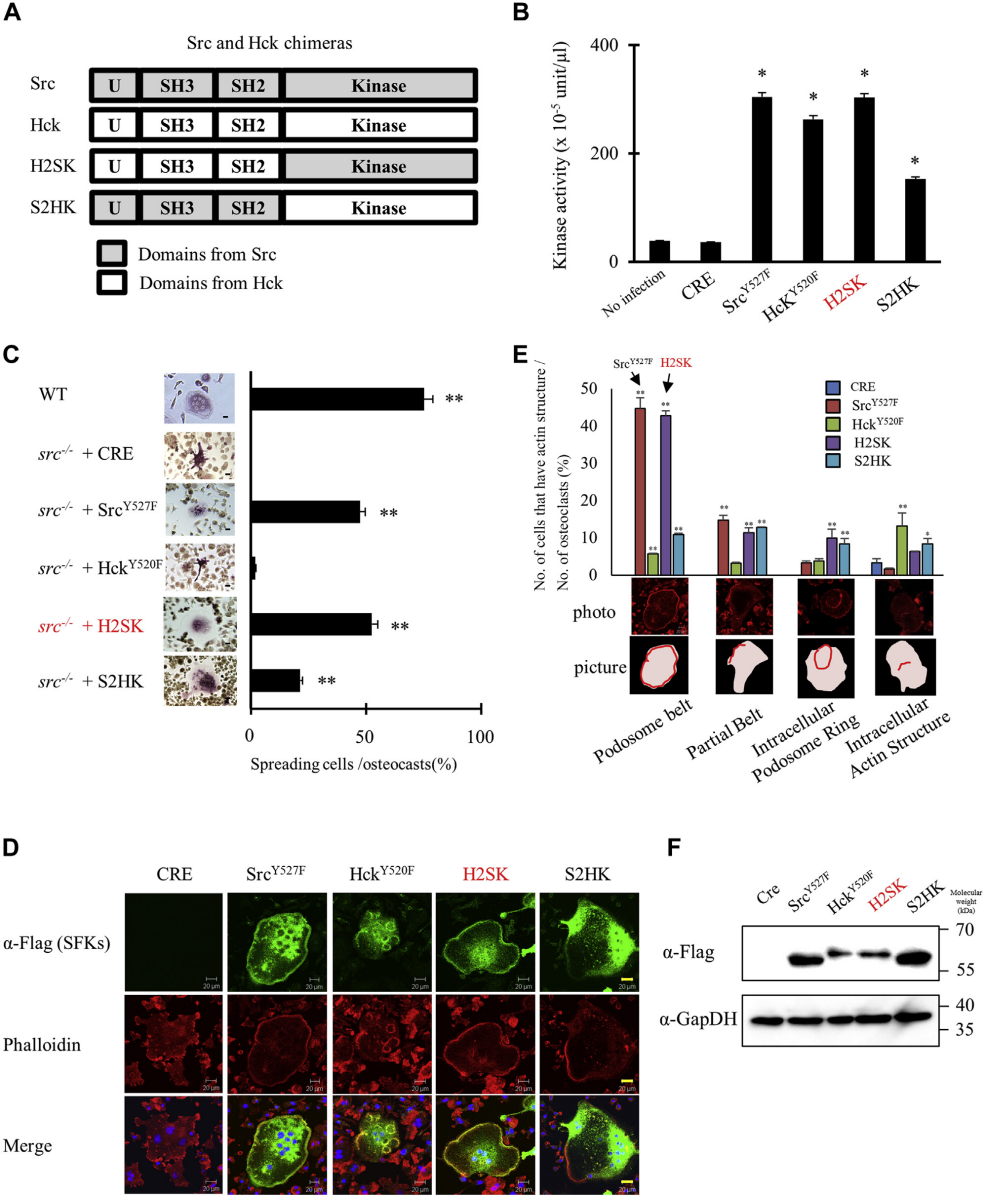
**The Src kinase domain was important for podosome organization.***A*, schema of Src and Hck chimeras. “U” indicates unique domain, “SH3” indicates SH3 domain, “SH2” indicates SH2 domain, and “kinase” indicates kinase domain. *B*, SYF cells were infected with Cre adenovirus (MOI 50) and adenoviruses (MOI 50) that expressed the indicated Src family kinases and chimeras were cultured for 2 days. Cells were lysed, and kinase activity was measured using a universal tyrosine kinase assay kit. (Mean ± SD; n = 4). ∗*p* < 0.01 *versus* SYF no infection. *C*–*E*, differentiated *src*^*−/−*^ osteoclasts were infected with Cre-expressing adenovirus and adenoviruses that expressed Src^Y527F^, Hck^Y520F^, or chimeras as indicated and then after a day, the culture was stained by TRAP (*C*) or immunostained by rhodamine phalloidin (*red*), anti-Flag/anti-mouse Alexa Fluor 488 (*green*) and DAPI (*blue*) (*D*). Osteoclasts and the cells having podosome belt were counted to obtain the ratio. A representative photo and a picture diagram (showing the cell body as *pink* and actin structure as a *red line*) are presented under the graph. (Mean ± SD; n = 4). ∗*p* < 0.05 *versus src*^*−/−*^ with Cre and ∗∗*p* < 0.01 *versus src*^*−/−*^ with Cre. *E* and *F*, *src*^*−/−*^ osteoclasts were infected cre and indicated SFK chimera adenovirus. The expression level of indicated proteins was determined by Western blotting analysis. MOI, multiplicity of infection; SFKs, Src family kinases; TRAP, tartrate-resistant acid phosphatase.

Hck^Y520F^ fails to significantly restore the spreading and podosome belt formation in *src*^−/−^ osteoclasts (Fig. 2). However, the absence of both Src and Hck results in significantly more severe osteopetrosis than the absence of Src alone (7), suggesting that some feature of Hck may be functionally redundant with certain Src domains. To explore the contributions of Src’s individual domains to its unique ability to promote podosome belt formation, we constructed chimeras of Src and Hck and expressed them in *src*^−/−^ osteoclasts. We hypothesized that the specialized SFK binding domains, especially the SH2 and SH3 domains, could determine the specificity of Src. We first swapped the SH2 and SH3 binding domains of Hck and Src to generate two complementary chimeras with the binding domains of one SFK and the catalytic domain of the other (Fig. 3*A*). Both chimeras had nonspecific kinase activity with a short peptide substrate, although the activity of the chimera with the Hck kinase domain had only about half the activity of the Src kinase domain chimera (Fig. 3*B*). Similar data were obtained by Western blotting analysis (Fig. S2).

Contrary to our hypothesis that the binding domains would provide Src’s functional specificity, we found that the Hck chimera with Hck SH2-SH3 and the Src kinase domain (H2SK) was comparable to Src itself in its ability to restore cell spreading, shape, and podosome belt formation in *src*^−/−^ osteoclasts when expressed at the same expression level as Hck^Y520F^. In contrast, the Src chimera with Hck kinase domain and Src SH2–SH3 (S2HK) only partially restored cell spreading and had much less of an effect on the podosome belt formation than Src although the expression level of S2HK was almost same (Fig. 3, *C*–*F,* Fig. S3). These results suggest that the Src and Hck SH2 and SH3 domains have comparable specificity for the target proteins that mediate the localization of Src during the induction of spreading and podosome belt formation, but the kinase domains differ in their ability to phosphorylate the proteins that play key roles in these processes.

### Src’s unique and SH3 binding domains are important for podosome organization

**Figure 4:**
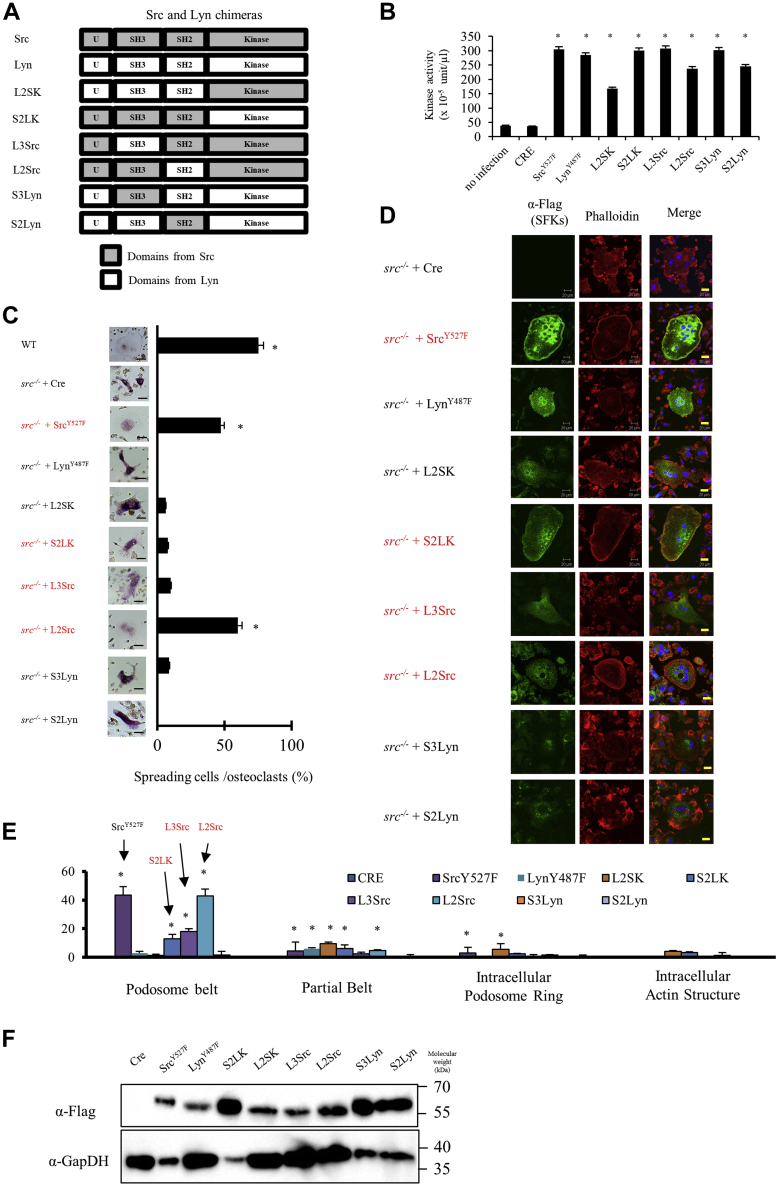
**Src unique and SH3 domains were important for podosome organization in osteoclasts.***A*, schema of Src and Lyn chimeras. *B*, SYF cells were infected with Cre adenovirus (MOI 50) and adenoviruses (MOI 50) that expressed the indicated Src family kinases and chimeras and cultured for 2 days. Cells were lysed, and kinase activity was measured using a universal tyrosine kinases assay kit. (Mean ± SD; n = 4). ∗*p* < 0.01 *versus* SYF no infection. *C*, differentiated *src*^*−/−*^ osteoclasts were infected with Cre adenovirus (MOI 50) and adenoviruses that express Src, Lyn, and chimeras as indicated (MOI 50), then after a day, the culture was stained by TRAP or immunostained by rhodamine phalloidin (*red*), anti-Flag/anti-mouse Alexa Fluor 488 (*green*), and DAPI (*blue*). *D*, osteoclasts and the cells having podosome belt were counted to obtain the ratio. (Mean ± SD; n = 4). ∗*p* < 0.01 *versus src*^*−/−*^ with Cre. *E*, osteoclasts and the cells having podosome belt were counted to obtain the ratio. *F*, *src*^*−/−*^ osteoclasts were infected cre and indicated SFK chimera adenovirus. The expression level of indicated SFKs was determined by Western blotting analysis. MOI, multiplicity of infection; SFK, Src family kinase; TRAP, tartrate-resistant acid phosphatase.

Despite the high level of Lyn expression, *src*^−/−^ osteoclasts do not spread to a round shape or form podosome belts. Moreover, overexpression of constitutively active Lyn in mature *src*^−/−^ osteoclasts did not restore cell spreading or podosome belt formation (Fig. 2). These results suggest that Lyn is largely unable to replicate Src activity in osteoclasts. Thus, replacing individual Src domains with the corresponding Lyn domains should at least partially disable Src function. We therefore constructed eight Src-Lyn chimeras to analyze the functional role of each Src domain (Fig. 4*A*).

Like the Src–Hck chimeras, the Src–Lyn chimeras retained kinase activity with both peptide and protein substrates although target proteins were different (Fig. 4*B*, Fig. S4). The replacement of either the Src kinase domain (S2LK) or the Src-binding domains (L2SK) with Lyn domains eliminated much of Src’s ability to restore spreading (Fig. 4*C*) or podosome belt formation (Fig. 4, *D* and *E*). The near-complete inability of the chimera with the Lyn SH2 and SH3 domains (L2SK) to promote spreading or podosome belt formation suggests that Src SH2 and/or SH3 domains are required for both activities of Src. On the other hand, the chimera of the Src SH2 and SH3 with Lyn kinase domain (S2LK) was about 10-fold more active in supporting belt formation than the complementary half-chimera, indicating that the Lyn kinase domain has some ability to phosphorylate Src target protein(s) that promote belt formation when properly localized by the Src-binding domains, but still at only 25 to 30% of the activity of full-length Src.

We further analyzed the contributions of the three Src-binding domains (unique, SH3, and SH2) by replacing one or two of them with the corresponding Lyn domains (Fig. 4, *B*–*F,* Fig. S5). Replacing only the SH2 domain of Src with the Lyn SH2 (L2Src) had little effect on either spreading or belt formation. In contrast, replacing the SH3 domain of Src with Lyn SH3 (L3Src) significantly reduced both spreading and belt formation, suggesting that Src specificity in osteoclast cytoskeletal organization depends in large part upon the SH3 domain.

### Phosphorylation of c-Cbl by c-Src promotes Src–Cbl interaction

**Figure 5:**
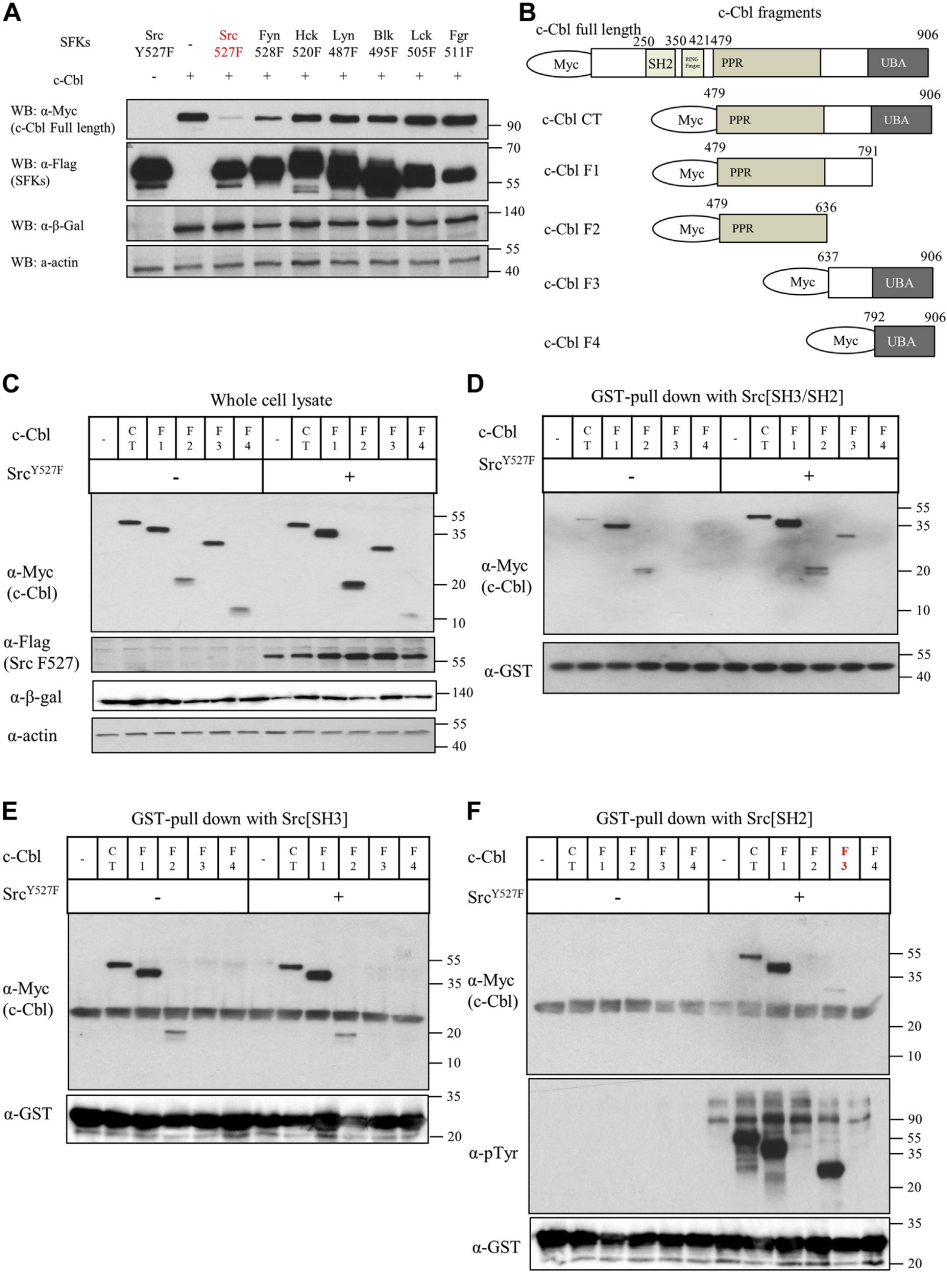
**c-Src bound to c-Cbl *via* an SH3–PPR interaction and phosphorylated c-Cbl tyrosines, creating binding sites for c-Src’s SH2 domain.***A*, SYF cells were transfected with myc-tagged c-Cbl and β-galactosidase. One day after transfection, cells were infected with adenoviruses expressing Flag-tagged Src family kinases with CRE adenovirus as indicated and cultured for 1 day. Cells were lysed, and the lysates were immunoblotted with antibodies against myc, flag, β-galactosidase (indicated as β-Gal) and actin. β-galactosidase indicates transfection efficiency. *B*, schema of truncated forms of c-Cbl. *C*–*F*, HEK293 cells were transfected with myc-tagged c-Cbl fragments with or without c-Src Y527F for 2 days. Transfected cells were lysed and immunoblotted with antibodies against myc, flag, and actin as indicated (*C*). Cell lysates were incubated with immobilized GST–Src[unique–SH3–SH2] (*D*), GST–Src[SH3] (*E*), and GST–Src[SH2] (*F*) domains for 1 h, and the beads were isolated and washed. Bound proteins were immunoblotted with anti-myc and anti-GST antibodies. Myc, Myc-tag; PPR, proximal proline-rich region; Ring finger, Ring finger domain; SH2, Src homology 2 domain; UBA, UBA/leucine zipper domain.

The major molecular mechanism by which c-Src functions in osteoclasts is the phosphorylation of c-Cbl, a ubiquitin E3 ligase that regulates podosome belt formation (17, 26, 27, 28). Thus, specificity of SFK binding to c-Cbl or SFK phosphorylation of c-Cbl may explain the molecular uniqueness of c-Src. We therefore determined whether this critical interaction was based upon the specificity of Src SH3 by examining the mechanism by which c-Src interacts and phosphorylates c-Cbl. As we reported earlier (29), coexpression of Src and c-Cbl induced c-Cbl and Src degradation, with c-Cbl levels reduced by more than 90% (Fig. 5*A*, Fig. S6). Fyn also induced degradation of c-Cbl by about 50% when Src was not co-overexpressed (Fig. 5*A*, Fig. S6). In contrast, other Src family kinases had little or no effect on c-Cbl level (Fig. 5*A*, Fig. S6). These results suggest that the Src-induced degradation of c-Cbl could contribute to the unique effect of Src on actin organization in osteoclasts.

c-Cbl’s PPR domain contains multiple sites that bind to the Src SH3 domain (27, 30). However, mutating the PPR motifs does not completely eliminate binding to Src (27), and it has been reported that mutating phosphorylated c-Cbl Tyr residues reduces c-Cbl’s binding to Src and other SFKs (31). To better understand the roles of c-Cbl SH3-binding and SH2-binding motifs in mediating c-Cbl’s interaction with c-Src, we made truncated fragments of the C-terminal half of c-Cbl (c-Cbl-CT) that eliminated one or more specific binding motifs (Fig. 5*B*) and overexpressed these five mutants with or without Src^Y527F^ in 293 cells (Fig. 5*C*, Fig. S7*A*). The binding of the fragments to Src domains was evaluated by pulldown assays using glutathione-*S*-transferase (GST)–Src constructs containing Src’s entire N-terminal half (unique, SH3, and SH2 domains, Fig. 5*D*, Fig. S7*B*), SH3 domain (Fig. 5*E*, Fig. S7*C*), or SH2 domain (Fig. 5*F,* Fig. S7, *D* and *E*). Consistent with earlier reports, c-Cbl-CT (aa 479–906), F1 (aa 479–791, lacking the UBA domain), and F2 (aa 479–636, lacking the C-terminal phosphorylated Tyr residues and UBA domain) bound to N-terminal Src and Src SH3 in the absence of coexpressed Src^Y527F^, whereas F3 (aa 637–906, lacking the PPR motifs) and F4 (aa 792–906, lacking both PPR domains and phosphorylated Tyr residues) did not. Coexpressing Src^Y527F^ to catalyze phosphorylation of the c-Cbl Tyrs increased the binding of N-terminal Src to c-Cbl-CT and F1 (and to a lesser degree F2). More importantly, Src-catalyzed phosphorylation induced the binding of N-terminal Src to F3, which lacks the SH3-binding PPR motifs, indicating that phosphorylation of one or more of the c-Cbl Tyrs creates a binding site for Src. Src-catalyzed phosphorylation had little or no effect on the binding of any of the fragments to Src SH3, suggesting that Src-catalyzed phosphorylation of c-Cbl Tyrs promoted binding to the Src SH2 domain. This was confirmed using GST-c-Src SH2 in the pull-down assay (Fig. 5*F*, Fig. S7*E*). c-Cbl CT, F1, and F3 were highly phosphorylated bound to GST-c-Src SH2 when Src^Y527F^ was coexpressed, but no binding was observed when c-Src^Y527F^ was absent (Fig. 5*F*). The binding of phosphorylated F3 was relatively weak, despite its high phosphorylation (compare the binding of CT and F3), suggesting that binding of the SH2 domain to phosphorylated c-Cbl is weak relative to the SH3–proline motif interaction. F2, which lacks the phosphorylated Tyrs, did not bind to Src SH2 whether c-Src^Y527F^ was coexpressed or not.

### Phosphorylation of c-Cbl Y731 and Y774 enhances Src interaction

**Figure 6:**
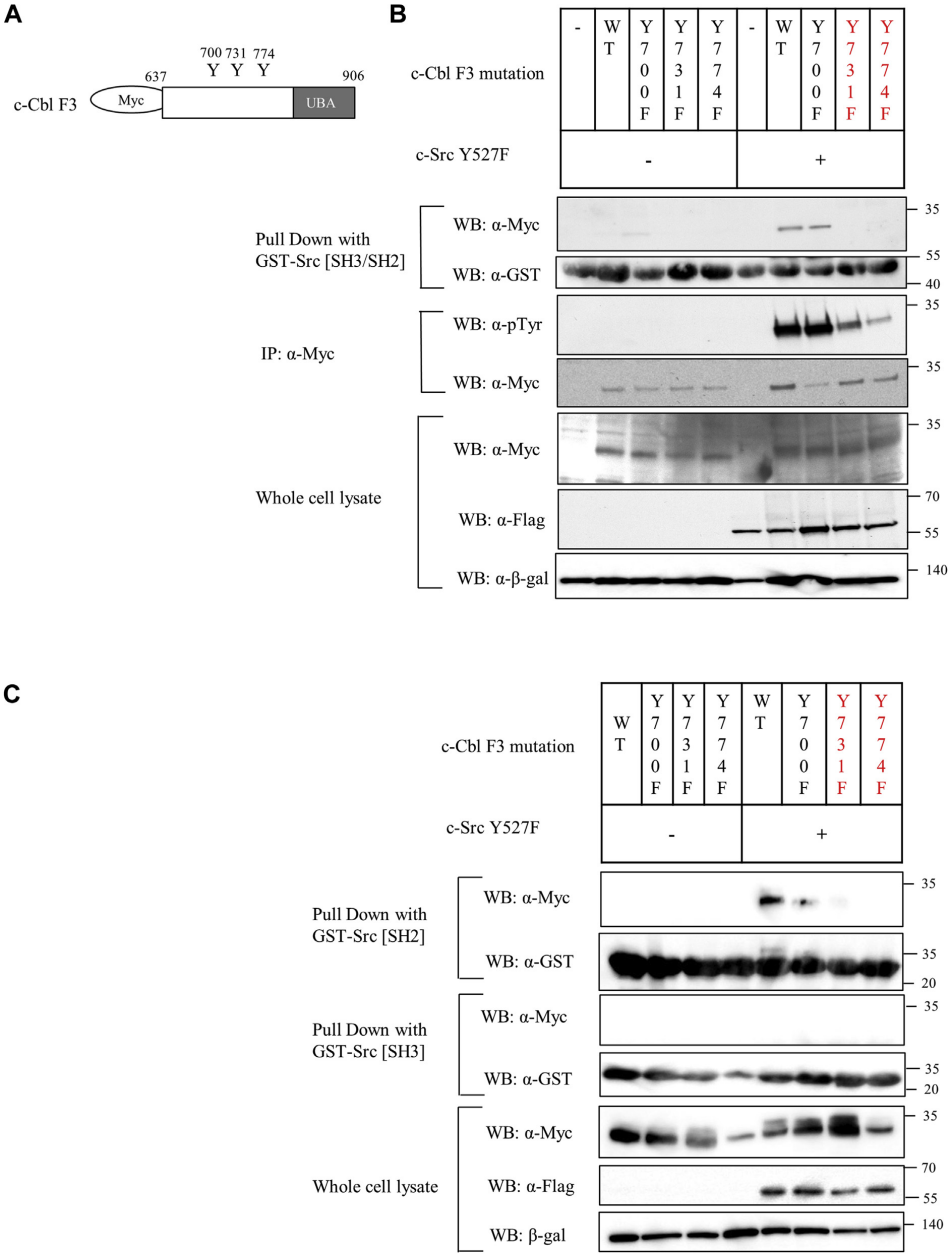
**Phosphorylation of c-Cbl**^**Y731**^**and c-Cbl**^**Y741**^**was important in binding to Src.***A*, schema of tyrosine site of c-Cbl fragment 3. *B* and *C*, c-Cbl fragment 3 and its point mutants (Y700F, Y731F, and Y774F) were overexpressed with or without Src^Y527F^ for 2 days. Cell lysates were incubated with GST–Src[SH3-SH2] domains (*B*) or GST–Src[SH2] (*C*) domain for 1 h, and the beads were isolated and washed. Bound proteins were immunoblotted with anti-myc and anti-GST antibodies as indicated. T antibodies are as indicated.

Three phosphorylated tyrosine residues (Tyr^700^, Tyr^731^, and Tyr^774^) have been reported to mediate the binding of multiple proteins to c-Cbl (32). We therefore mutated each of these residues to phenylalanine in c-Cbl F3 (Fig. 6*A*) and examined the effect on the binding of the Src SH2 domain. As expected, neither F3 nor the mutants bound to GST–Src [SH3/SH2] in the absence of coexpressed Src^Y527F^ (Fig. 6*B*, the left side of the top panel, Fig. S8*A*). When Src^Y527F^ was coexpressed with the c-Cbl F3 constructs, F3 and F3^Y700F^ were equivalently phosphorylated and bound to GST–Src [SH3/SH2] (Fig. 6*B*, the right side of the top panel, Fig. S8*A*). In contrast, F3^Y731F^ and F3^Y774F^ were much significantly less phosphorylated and failed to detectably bind to GST–Src [SH3/SH2] (Fig. 6*B* second panel, Fig. S8*B*), suggesting that phosphorylation of both Tyr^731^ and Tyr^774^ can enhance c-Cbl binding to c-Src. Next, we sought to determine which domain of Src is involved in binding to phosphorylated c-Cbl F3. GST–Src SH3 domain did not bind to both nonphosphorylated and phosphorylated c-Cbl F3 (Fig. 6*C*, second panel). On the other hand, GST–Src SH2 bound to F3 and F3^Y700F^ (Fig. 6*C*, the top panel, Fig. S8*D*). These results suggest that Tyr^731^ and Tyr^774^ can enhance c-Cbl binding to the c-Src SH2 domain.

## Discussion

The Src tyrosine kinase plays an essential role in osteoclast bone resorbing activity, promoting the formation of the sealing zone and ruffled border and organization of F-actin into the peripheral podosome belt (2, 17). Of the eight SFKs expressed in mammals, only Src is required for normal bone resorption (33). Although c-Src is expressed in many tissues, osteopetrosis is the only phenotype of *src*^−/−^ mice (34), suggesting that osteoclasts are the only cells in which the function of Src is not efficiently compensated by other SFKs. However, eliminating both Src and Hck results in a more severe osteopetrosis than the single Src deletion (7), suggesting that Hck might partly compensate for the absence of Src in osteoclasts. In this study, we sought to identify the basis of the unique function of c-Src in osteoclasts by testing two hypotheses that (1) other SFKs may be expressed in osteoclasts at much lower levels than Src and fail to compensate for the absence of Src in part due to insufficient expression and (2) osteoclasts require a specific Src function(s) that other SFKs, although expressed in osteoclasts, cannot perform. Our results indicate that both factors contribute to the unique requirement for Src in osteoclasts.

We first determined SFK expression in WT and *src*^*−/−*^ osteoclasts. In the *src*^−/−^ cells, only Lyn and Hck expression increased, to about 400% and 80% of WT Src expression, respectively, suggesting that neither Lyn nor Hck are capable of performing critical osteoclast-specific Src functions, despite higher or equivalent levels of expression in Src−/− osteoclasts. Indeed, overexpressing constitutively active Lyn in WT osteoclasts had no effect on podosome belt formation, indicating that the extremely high level of Lyn in the *src*^−/−^ cells did not contribute to the *src*^−/−^ osteoclast phenotype.

The low expression of Fyn, Yes, Blk, Lck, and Fgr in the *src*^−/−^ osteoclasts left open the possibility that one or more of these SFKs could act in Src’s place if expressed at WT Src expression levels.

There are however some limitations to this study. To identify the specific domains of Src responsible for the osteoclast phenotype, we used open conformation, constitutively active, SFK constructs. The rationale for this approach is that all SFKs will be in an open and active conformation, independent of specific regulatory protein interactions. For example, SFKs can be inactivated upon C-terminal tyrosine phosphorylation by the C-terminal Src kinase (CSK) (35). SFKs phosphorylation by CSK leads to intramolecular interactions between the C-terminal tail and the SH2 domain, which results in a closed inactive conformation, rendering our identification of specific domains impossible. The inhibition of SFKs by CSK is itself dependent on multiple binding partners unique to specific SFKs. For example, Src regulation by CSK is dependent on the Cbp complex (36, 37), whereas Lyn and Lck regulation by CSK is dependent on the Lime complex (38). Thus, open and constitutively active constructs eliminate negative regulation of SFKs by CSK complexes. WT SFK constructs will thus be at varying states of activation. This however does not alter the interactive and regulatory properties of each domain, a key part of our strategy.

The use of constitutively activated forms does however limit the physiologic relevance of our observations. Ectopic expression of active forms, in other systems, has been shown to strikingly alter cell morphology and transformation (39, 40). Although we did not observe such effects, we should be mindful of the artificial nature of overexpression studies in general. Nonetheless, artificial hybrid chimeric constructs are necessary, and provide useful molecular information within the context of this *in vitro* study aimed at identifying the domains that confer to Src unique function(s) in osteoclasts. Indeed, the functional rescue of *src*^−/−^ podosome formation and osteoclast activity by constitutively active constructs in this study in a manner similar to WT cells (14) supports this view. These data will allow further *in vivo* studies to extend our understanding of SFK biology and its role in osteoclast function.

It is intriguing that, of these SFKs, only Fyn significantly improved podosome belt formation. WT Fyn was also able to rescue podosome belt formation in a manner similar to the constitutively active form (Fig. S9). Thus, Fyn, which is about 60% homologous to Src, is capable of performing the critical Src activities that promote osteoclast function but fails to do so in *src*^−/−^ osteoclasts because of insufficient level of expression in osteoclasts. To support this, the phosphorylation patterns of Src and Fyn are quite similar (Fig. S10). In contrast, the other SFKs lack Src’s ability to promote osteoclast bone-resorbing activity, in particular, podosome belt formation, even when expressed at levels comparable with Src.

Drawing on our findings that Lyn and Hck are relatively highly expressed in the *src*^−/−^ osteoclasts but fail to substitute for Src in promoting podosome belt formation, we explored the contributions of Src’s individual domains to the kinase’s unique function(s) in osteoclasts by exchanging Hck or Lyn domains for the homologous Src domains.

We first compared the binding and catalytic functions of Hck and Src by exchanging the kinase domains. We had predicted that the specificity of Src would reside primarily in the binding domains (unique, SH3, and SH2) in the N-terminal half of the proteins. Instead, we found that it was the kinase domains that differed in their ability to restore podosome belt formation (Fig. 3). This indicates that the Hck-binding domains are equivalent to the homologous Src domains in terms of performing these functions. Thus, the Src- and Hck-binding domains may have similar specificity for the binding partners that mediate the localization of Src during the induction of podosome belt formation, but the kinase domains differ in their ability to phosphorylate the key substrate proteins in these processes. The partial restoration of podosome belt formation by the S2HK chimera may explain why Hck deletion increases the osteopetrosis of the double Src–Hck KO mice, but the failure of overexpressed Hck to improve podosome belt formation suggests that Hck may also play some role unrelated to Src.

We next replaced Src domains with the homologous Lyn domains to effectively eliminate the function of the Src domains, allowing us to test the function of each Src domain in turn. In contrast to the Src–Hck hemichimeras, neither of the Src-Lyn hemichimeras retained the ability of Src to restore podosome belt formation, indicating that both the kinase domain and the binding domains contribute to Src’s ability to promote podosome belt formation. Interestingly, although both chimeras lost more than 90% of the ability to induce podosome belt formation, the chimera with the Lyn kinase domain (S2LK) was about 30% as active as Src at inducing podosome belt formation, suggesting that the substrate selectivity of the Lyn kinase domain is more Src like than the Hck kinase domain in the context of podosome belt formation.

Replacing the Src SH3 domain by Lyn SH3 domain (L3Src) eliminated 75 to 85% of the induction of podosome belt formation, and the effects were additive. When both the unique and SH3 domains were replaced, the chimera failed to improve podosome belt formation at all. In contrast, replacing the Src SH2 domain did not affect Src’s ability to restore podosome belt formation, indicating that specific localization of Src related to the regulation of podosome belt formation is mediated by the unique and SH3 domains. Like the Lyn kinase domain, the Lyn SH3 domain retained some ability to induce podosome belt formation (∼40% of the Src SH3 activity), indicating that while both of Src’s unique and SH3 domains contribute to directing Src to signaling complexes that promote belt formation, the unique domain is more important than the SH3. These results indicate that the uniqueness of Src in osteoclasts is based on both molecular function and level of expression. The pattern of tyrosine-phosphorylated proteins is quite different between each other (Fig. S4). It is expected that the comparison of these phosphorylated proteins will reveal the target molecules of Src in podosome formation.

For further analysis of Src’s unique function, we focused on c-Cbl, an E3 ubiquitin ligase that is an essential downstream protein target of Src in podosome belt formation (17, 26, 27, 28). Src binds to c-Cbl’s PPR domain and phosphorylates c-Cbl’s tyrosine residues (10, 41). In turn, phosphorylated c-Cbl binds to Src SH2 domains, further activating Src (12, 17) and leads to ubiquitylation and degradation of both Src and c-Cbl (29, 42). Here, we found that Fyn induces a weaker effect on c-Cbl than Src and that Hck, Lyn, and other SFKs do not reduce c-Cbl levels. It is presumed that phosphorylation of c-Cbl does not occur because of a different functionality in the catalytic domain of Hck, given our data showing the contribution of Src kinase domain to its specificity. These suggest that c-Cbl is a target of Src’s unique function.

We showed that Src SH3 domain binds to the aa 479 to aa 636 region of c-Cbl. Consequently, Src phosphorylates Tyr 731 and Tyr 774 of c-Cbl. Phosphorylated c-Cbl binds to Src’s SH2 domain. Src can also bind to c-Cbl outside of the PPR domain because constitutively activated Src phosphorylated the c-Cbl F3 (aa 637 to aa 906) fragment, which does not contain the PPR domain. However, the binding affinity of c-Cbl F3 fragment for Src SH2 is weaker than the c-Cbl F1 fragment. Although Src SH3 domain does not directly bind to the c-Cbl F3 fragment, c-Cbl F3 is phosphorylated when coexpressed with Src^Y527F^. c-Cbl F3 may be phosphorylated by Pyk2 or other kinases present in a complex with Src and c-Cbl (43). This suggests that c-Cbl PPR is important for sufficient binding of c-Cbl and Src SH2 domain.

Downstream of Src, phosphorylated c-Cbl also binds and regulates phosphoinositide 3-kinase and small G-proteins such as Rac and Rho to organize actin filaments and podosomes (6, 17, 44, 45). Furthermore, phosphorylated c-Cbl constructs stabilize microtubules by binding to CIN85, regulating microtubule assembly and degrading histone deacetylase 6 (10, 28). Thus, the Src–Cbl pathway is essential to induce podosome belt formation.

In conclusion, compared with Hck or Lyn, Src has a unique function in podosome belt formation of osteoclasts because of its distinct kinase and SH3 domains in the chimera constructs. Moreover, although Src and Fyn do have redundant functions in podosome belt formation, the regulation of Src expression and the effect of Src on c-Cbl is different from that of Fyn.

## Experimental procedures

### Animals and cells

*src*^*+/−*^ mice (34) were purchased from The Jackson Laboratory (MA). *src* ^*+/−*^ mice were crossed each other to obtain WT and *src*^*−/−*^ mice. *src*^*−/−*^ mice were fed mill food after weaning because of loss of tooth eruption. All mice were housed at the Harvard Medical School, and experimental protocols were approved by the Harvard Institutional Animal Care and Use Committee.

*Src*-, *yes*-, and *fyn*-deficient fibroblasts (SYF cells) and 293 cells were purchased from the American Type Culture Collection and cultured in Dulbecco's modified Eagle's medium from Sigma-Aldrich containing 10% fetal bovine serum.

### Reagents

Anti-Src antibody was obtained from Merck Millipore. Anti-phosphotyrosine (pTyr-100) antibody, anti-pY731 c-Cbl antibody, and anti-phospho-cortactin antibody (pY421) were obtained from Cell Signaling Technology. Anti-c-Cbl antibody was obtained from Santa Cruz Biotechnology.

### Plasmids and adenovirus

Src and Lyn constructs were provided by Dr Clifford Lowell (University of California–San Francisco, CA). Fyn, Hck, Blk, Lck, and Fgr cDNAs were bought from Open BioSystems.

Constitutively active Src, Fyn, Hck, Lyn, Blk, Lck, and Fgr were generated by using the QuikChange Site-Directed Mutagenesis Kit from Agilent technologies with following primers: Src–5′- ttacgtccactgagccacagttccagcccggggagaacc -3′, Fyn–5′- tacggccacagagccccagtttcagcccggtgaaaacct -3′, Hck–5′- tacacggccactgagagccagtttcagcagcagccttga -3′, Lyn–5′- atacagccacagaagggcagtttcagcagcaaccgtag -3′, Blk–5′- acacagccacggagggccaatttgagctgcagccctag -3′, Lck–5′- tcacagccacagagggccagttccagccccagccttga -3′, Fgr–5′- cacctccacagaaccacagttccagcctggagaccagac -3′. Src chimeras were generated with PCR. Src kinase domain was amplified by PCR with the Hck or Lyn SH2 domain sequence containing the forward primer. Unique, SH3, and SH2 domains (N terminus) of Hck and Lyn were also amplified by PCR with Src kinase domain sequence containing the reverse primer. Subsequently, the Src kinase domain and N terminus of Hck or Lyn were connected by PCR. All kinase domains of Src and Hck and Lyn chimera constructs were cloned from constitutively active SFKs.

Constitutively active Src family kinases and Src chimeras added Flag-tag at the C terminus and inserted to the adenovirus vector (pAxCALNLwtit2) using an Adenovirus CRE/LoxP kit from Takara. Adenovirus was generated in 293A cells as described previously (37, 46). Titers of the viruses were determined using a modified point assay.

### Osteoclast differentiation *in vitro*

Spleen cells were isolated from 4- to 6-week-old WT or *src*^−/−^ mice and cultured under 5% CO_2_ at 37 °C in alpha modification of Eagle's minimum essential medium from Sigma-Aldrich supplemented with 10% fetal bovine serum, penicillin (100 U/ml), and streptomycin (100 μg/ml) with human macrophage colony-stimulating factor (30 ng/ml) from R&D Systems Inc for 3 days, and then, the cells (100,000 cells/well, in a 24-well plate) were replated on culture plates from Corning, coverslips (Fisher Scientific), or dentin slices and cultured with human soluble RANKL (100 ng/ml) from R&D Systems Inc for 5 days. Cells were infected with adenoviruses expressing cre recombinase (CRE) (multiplicity of infection [MOI] 50) and Src family kinases or chimeras (MOI 50) on day 4 after replating and culture for 1 day.

### TRAP staining

Cells were fixed with 3.4% formaldehyde for 10 min at room temperature (RT). Fixed cells were treated with 1:1 (vol/vol) ethanol/acetone for 1 min and washed by water. Cells were incubated in 0.1 M sodium acetate buffer, pH 5, containing 50 mM sodium tartrate, 2 mM Naphthol AS-MX phosphate (Sigma-Aldrich), and 2 mM Fast Red Violet LB salt (Sigma-Aldrich) at 37 °C for 15 min. TRAP-positive multinuclear cells (>3 nuclei) were considered as osteoclasts. The area of an osteoclast was measured with ImageJ (the National Institutes of Health). Spreading cells were defined and counted as cells with a round shape and 2 times or more of the cytosol area as the nuclear area. For image scoring, at least 300 cells/well (4 wells/group) of randomly selected osteoclasts were examined by two or more independent experimentalists. Evaluation was performed in a double-blind manner.

### Reverse-transcribed PCR and quantitative PCR

Total RNA was harvested from cultured WT or *src*^−/−^ osteoclasts using a QIAprep Spin Miniprep kit (Qiagen). After denaturation of total RNA at 70 °C for 10 min, cDNA was synthesized with oligo (dT) primer and Superscript II from Thermo fisher scientific Inc. qPCR was performed using an iCycler (Bio-Rad Laboratories). qPCR amplification was performed using the following primers specific for mouse SFKs: Src–sense primer, 5′- CATGTCTGCTCAGATCGCTT -3′; antisense primer, 5′- CACTTTGCACACCAGGTTCT -3′, Yes–sense primer, 5′- TGAGGCTGCTCTGTATGGTC -3′; antisense primer, 5′- CCCGCTCTACTTGTTCCAAT -3′, Fyn–sense primer, 5′- TACGTGGCTCCAGTTGACTC -3′; antisense primer, 5′- GTAGGCACCTTTGGTGGTTT -3′, Lyn–sense primer, 5′- TCACCGAGTTCATGGCTAAG -3′; antisense primer, 5′- TGTAGTTCTTCCGCTCGATG -3′, Hck–sense primer, 5′- CAGGAACTCGTGCTCCACTA -3′; antisense primer, 5′- TTCTCCATCTGGAGGGATTC -3′, Blk–sense primer, 5′- GAGGCAGGTCAGTGAGAAGG -3′; antisense primer, 5′- GTCCTGGTTAGGAGATGGTGG -3′, Lck–sense primer, 5′- TGCAAGATTGCAGACTTTGG -3′; antisense primer, 5′- GACCGTGGGTGACAATCTCT -3′, Fgr–sense primer, 5′- CTTCGTCCGTCTTTCCTCAG -3′; antisense primer, 5′- AACTTCTCGCCTTTGGTGAA -3′. Isolated transcripts were quantitated on iCycler IQ (Bio-Rad) using SYBR green. To quantify the SFKs’ expression level, we prepared 20,000 pg/ml, 4000 pg/ml, 800 pg/ml, 130 pg/ml, and 65 pg/ml of each SFK plasmid and determined a standard curve. Absolute expression value of SFKs were calculated using the standard curve.

### Immunofluorescence

Osteoclasts were cultured on coverslips for 5 days as described above. Cells were fixed in 3.4% formaldehyde for 10 min at RT. Fixed cells were permeabilized with ice-cold acetone and 0.2% Triton-X containing phosphate buffered saline (PBS) and incubated with 1% bovine serum albumin-PBS for 1 h. Cells were then stained with monoclonal anti-Flag M2 (Sigma-Aldrich), anti-mouse Alexa Fluor 488 (Thermo fisher scientific Inc), rhodamine phalloidin, and TO-PRO-3 (Invitrogen) (17, 37). Images were obtained using a confocal microscope LSM510 (Carl Zeiss). We considered podosomes surrounding >80% of osteoclasts as podosome belts. Podosomes surrounding <79% of osteoclasts were classified as partial belts. Podosomes forming a ring shape not at the cell periphery but at a more inner region within the osteoclast were classified as an intracellular podosome ring. Podosomes forming an undefined shape in the osteoclast were classified as intracellular actin structures. For image scoring, at least 300 cells/well (4 wells/group) of randomly selected osteoclasts were examined by two or more independent experimentalists. Evaluations were performed in a double-blind manner.

### Tyrosine kinase assay

1,000,000 SYF cells were cultured on 6-well plates and infected with adenoviruses expressing CRE (MOI 50) and Src family kinases or chimeras (MOI 50) as indicated. After 2 days culture, cells were harvested and lysed. Lysates were diluted 20-fold, and 40-μl samples were put into assay plates and measured by using the Universal Tyrosine Kinase Assay Kit (MK410, Takara) according to manufacturer’s instructions. One unit (U) of the enzyme is defined as the amount needed to incorporate 1 pmol of phosphate into the synthetic substrate (KVEKIGEGTYGVVYK) in 1 min.

### Western blotting

The cells were washed two times with ice-cold PBS and solubilized in the lysis buffer containing 20 mM Hepes (pH 7.4), 150 mM NaCl, 1 mM EGTA, 1.5 mM MgCl_2_, 10% glycerol, 1% Triton X-100, 10 μg/ml aprotinin, 10 μg/ml leupeptin, 1 mM 4-(2-aminoethyl)-benzenesulfonyl fluoride hydrochloride, and 0.2 mM sodium orthovanadate. The lysates were centrifuged for 20 min at 4 °C at 16,000*g*. The supernatants were boiled with 3xSDS sample buffer containing 0.5 M β-mercaptoethanol. Proteins were separated by SDS-PAGE, transferred to nitrocellulose membranes, immunoblotted with corresponding antibodies, and visualized with horseradish peroxidase–coupled anti-mouse or anti-rabbit IgG enhancement by Amersham ECL Western blotting detection kits provided from GE Healthcare Life Science. The expression levels of protein bands were determined with ImageJ.

### Data analysis and statistics

Statistical significance of differences between groups was analyzed by one-way ANOVA followed by a post hoc test. Differences were considered to be statistically significant if *p* < 0.05. Data are expressed as the mean values ± SD of the mean (mean ± SD; n = the number of culture wells). All experiments were performed at least twice independently and obtained similar data.

## Data availability

All the data are in this article.

## Supporting information

This article contains supporting information.

## Conflict of interest

The authors declare that they have no conflicts of interest with the contents of this article.
