# Supplementary material for: Characterization of unique functionalities in c-Src domains required for osteoclast podosome belt formation
Source: J Biol Chem. 2021 May 18;296:100790. doi: 10.1016/j.jbc.2021.100790 (PMC8196221; doi:10.1016/j.jbc.2021.100790)
Supplement: Supplemental Figures S1–S10 [file mmc1.pdf]

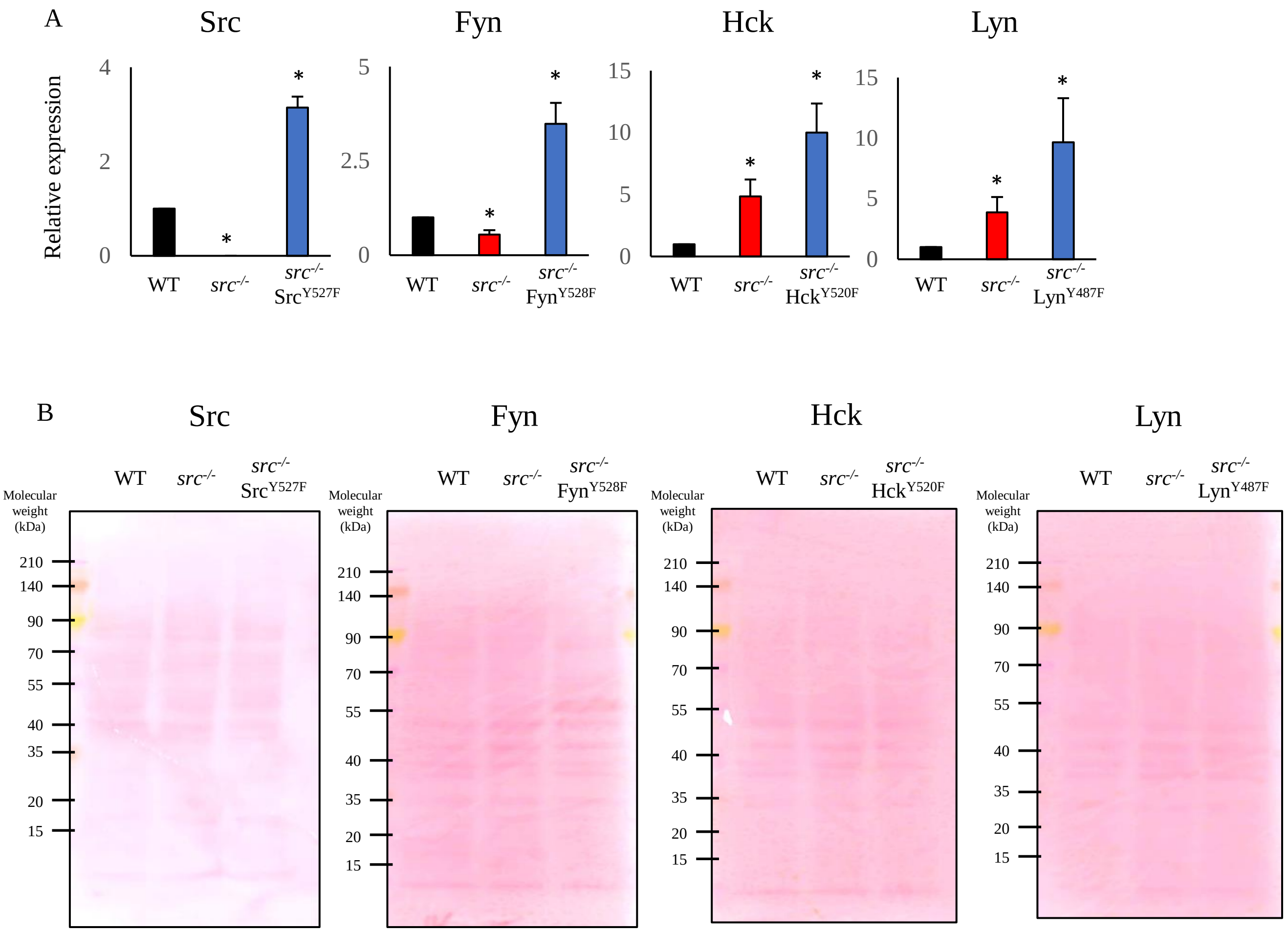


Supplemental Figure 1

(A) The expression level of each protein from Fig.2D was measured with Image J and normalized to the level of actin. The relative ratio compared to wild type (WT) was averaged from three independent experiments. Similar results of endogenous protein expression level with Fig.1A were obtained. Overexpression of each SFK was confirmed. (mean $\pm$ SD; n = 3). * denotes $P < 0.01$ vs WT. (B) Ponceau S staining of the membranes shown in Fig.2D showing equal loading of proteins.

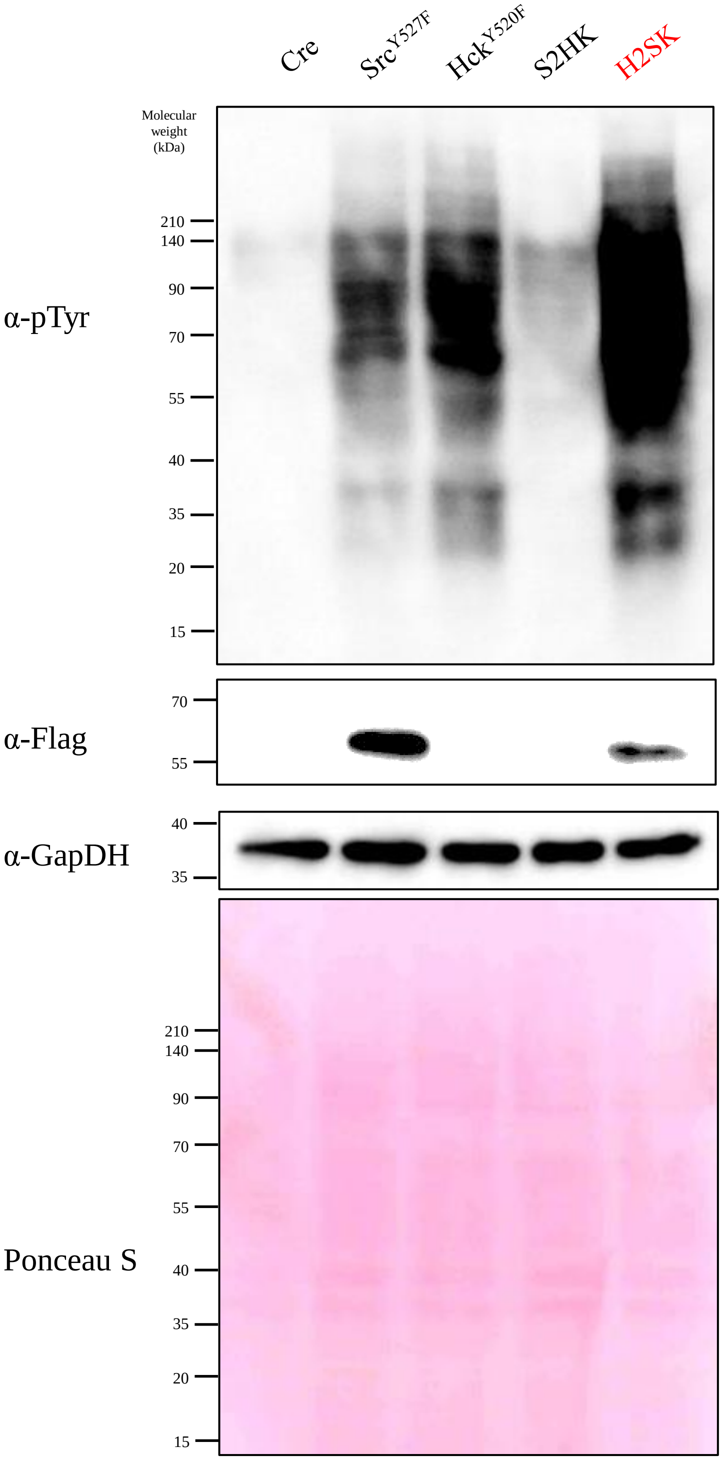


Supplemental Figure 2

Src$^{-/-}$ osteoclasts were infected with Cre adenovirus (M.O.I. 50) and adenoviruses (M.O.I. 50) that expressed the indicated Src family kinases and chimeras and cultured for 2 days. Cells were lysed and Tyrosine phosphorylation was examined by western blotting analysis. Similar to Fig 3B, kinase activity of S2HK is weaker than the other constructs.

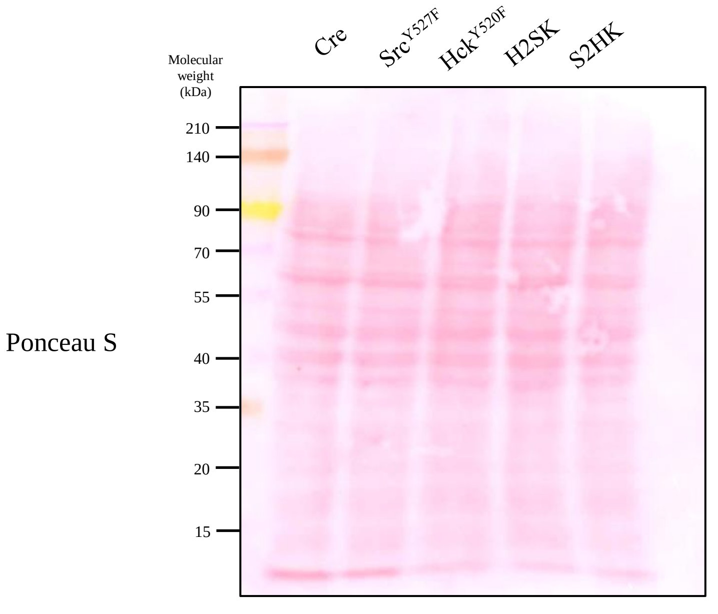


Supplemental Figure 3
Ponceau S staining of the membranes shown in Fig.3F. Equal amounts of proteins were loaded.

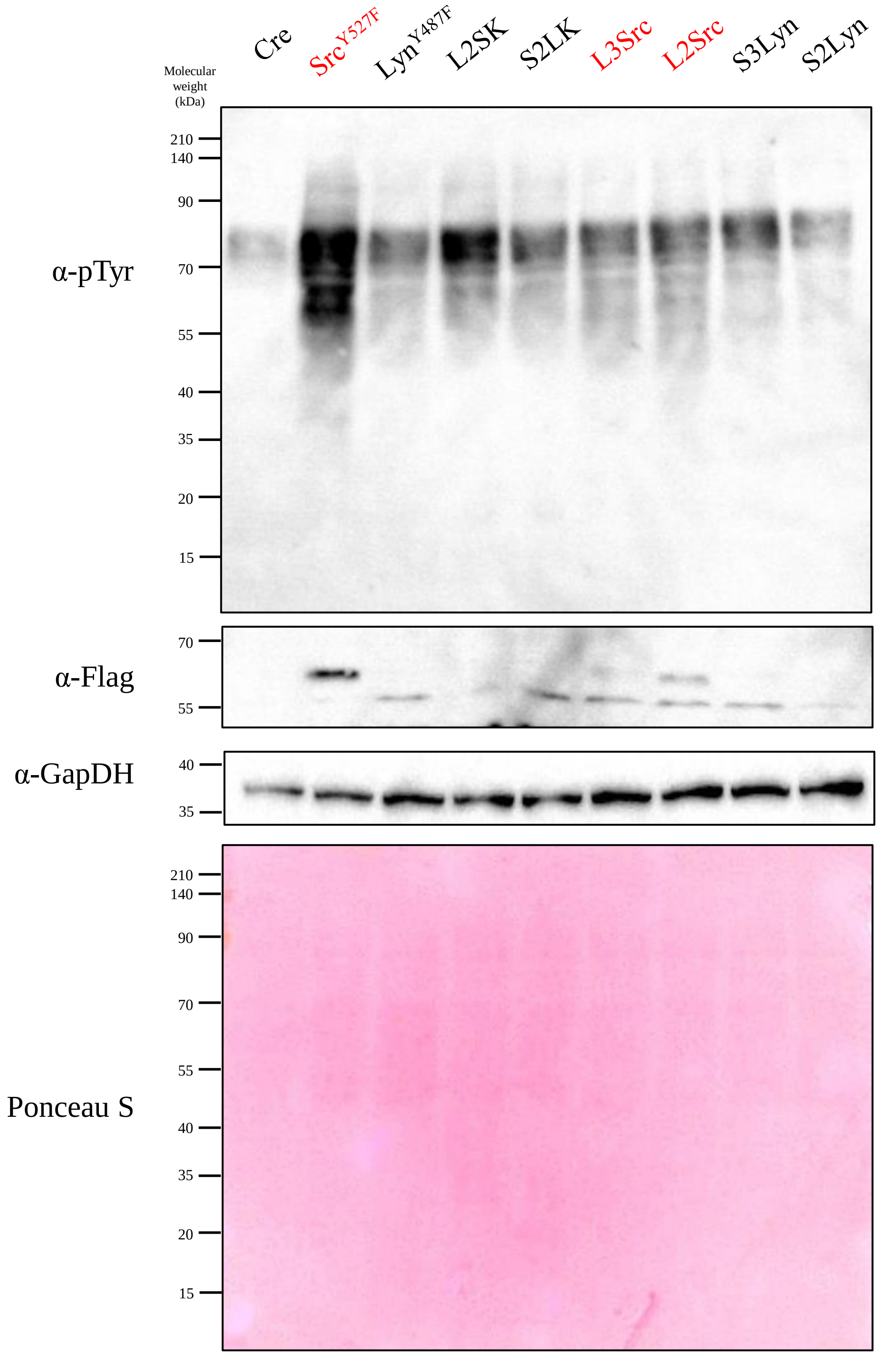


Supplemental Figure 4

Src$^{-/-}$ osteoclasts were infected with Cre adenovirus (M.O.I. 50) and adenoviruses (M.O.I. 50) that expressed the indicated Src family kinases and chimeras and cultured for 2 days. Cells were lysed and Tyrosine phosphorylation was examined by western blotting analysis.

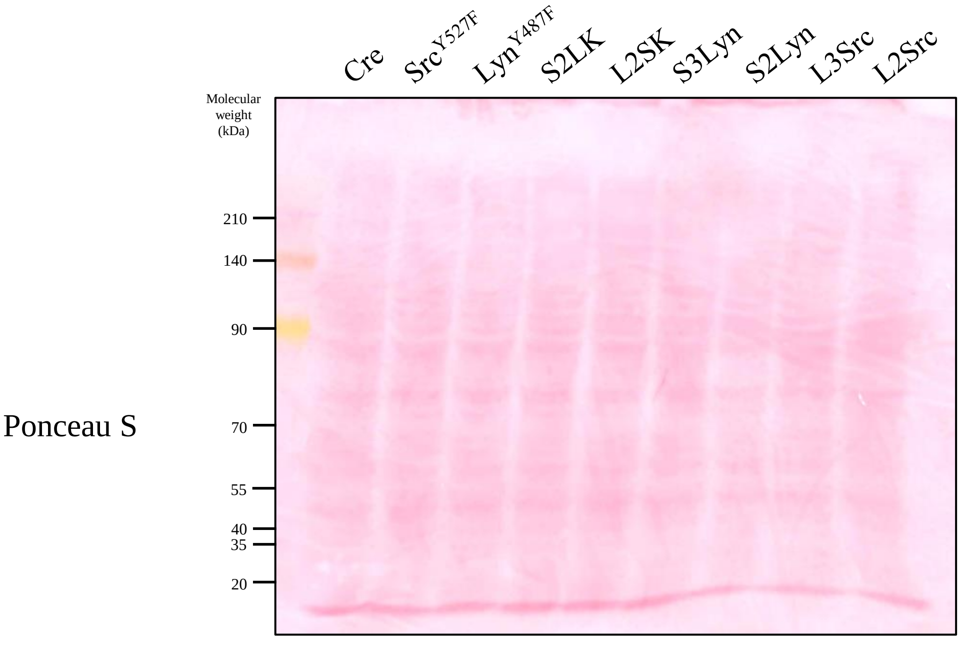


Supplemental Figure 5
Ponceau S staining of the membranes shown in Fig.4F. Equal amounts of proteins were loaded.

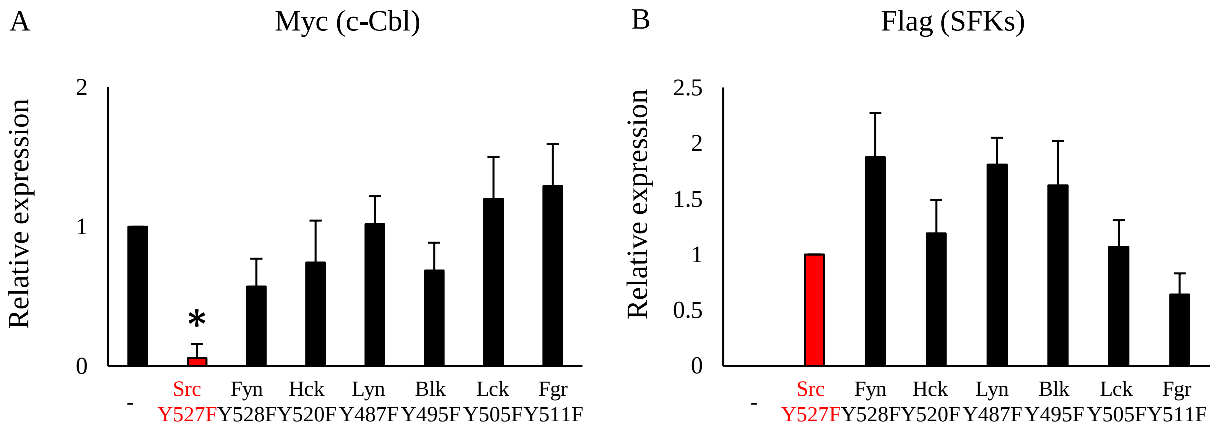


Supplemental Figure 6

(A, B) The expression level of each protein shown in Fig.5A was measured with Image J and normalized to the level of β-Gal. (A) The ratio vs SFK negative (-) was averaged from three independent experiments. (mean ± SD; n = 3). * denotes $P < 0.01$ vs no SFKs. (B) The ratio vs $Src^{Y527F}$ of these proteins was averaged from three independent experiments. (mean ± SD; n = 3).

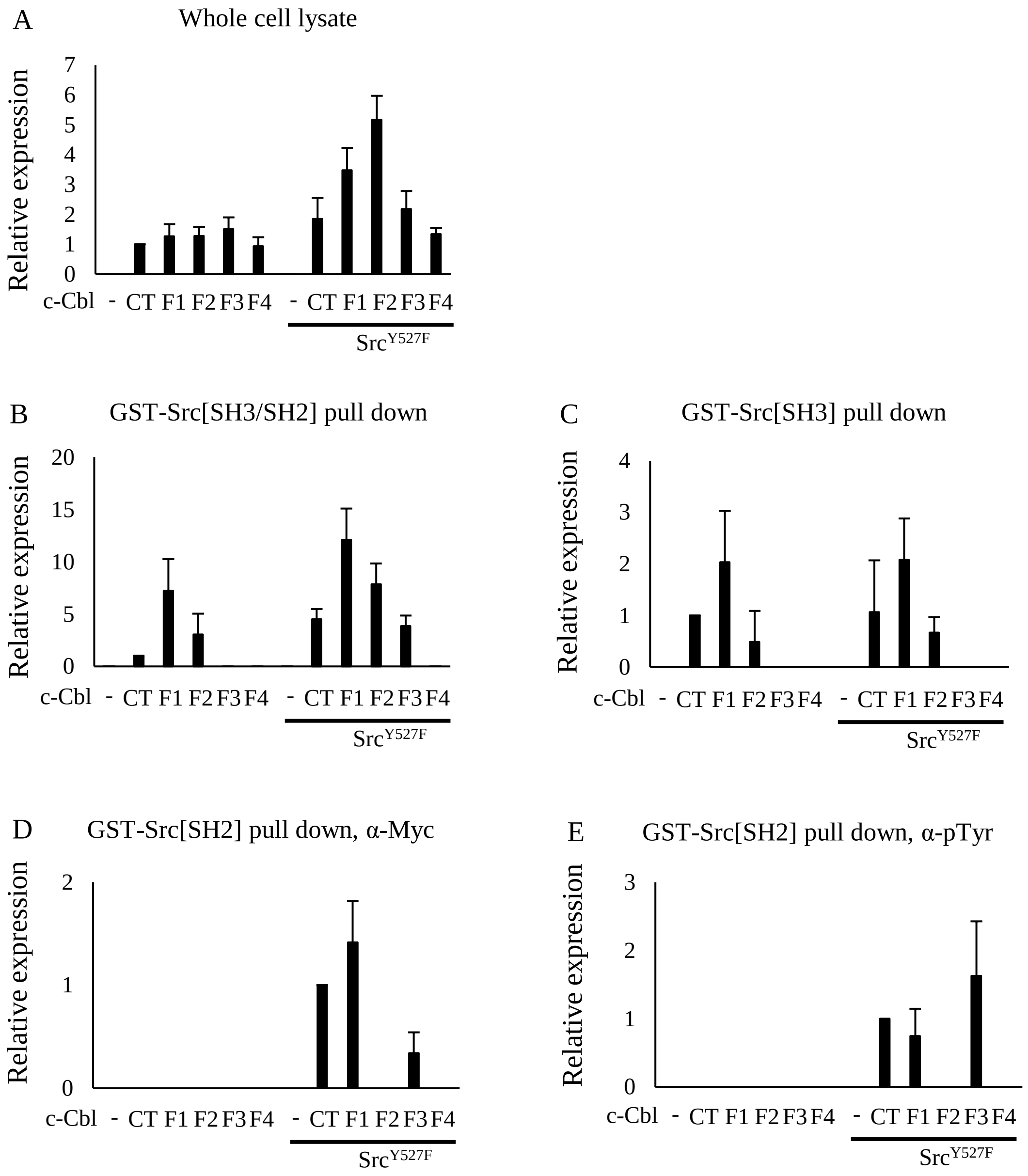


Supplemental Figure 7

Expression levels of the proteins shown in Fig.5C, D, E and F were measured with Image J. The expression levels of c-Cbl fragments (A, B, C, D) or phospho tyrosine (E) were normalized by β-Gal (A) or GST (B, C, D, E). The ratio vs c-Cbl CT without $Src^{Y527F}$ (A, B, C) or c-Cbl CT with $Src^{Y527F}$ (D, E) was averaged from three independent experiments. (mean ± SD; n = 3).

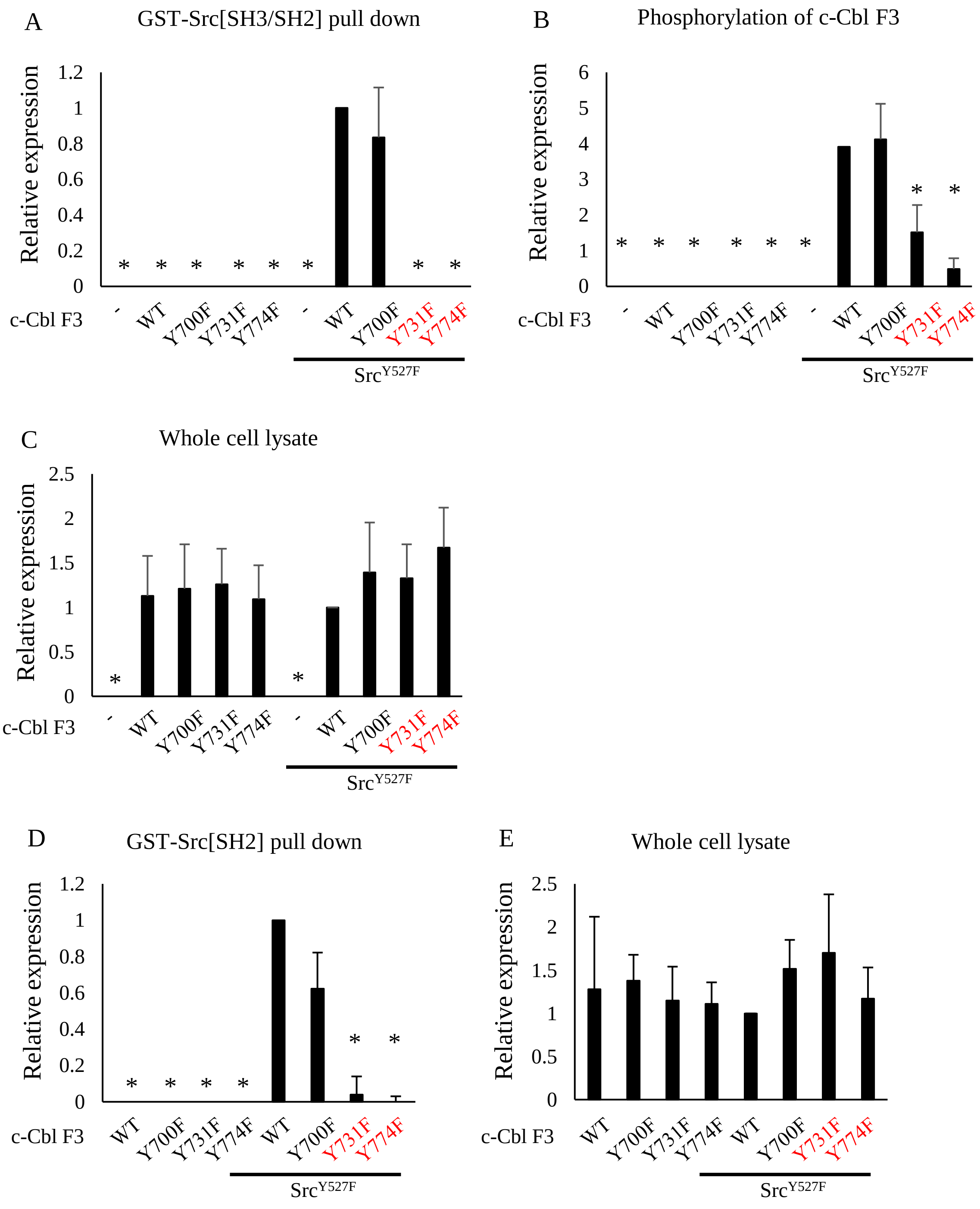


Supplemental Figure 8

Expression levels of the proteins shown in Fig.6 B, C were measured with Image J.

Expression levels of c-Cbl F3 fragments (A, C, D, E) or phospho tyrosine (B) were normalized by GST (A, D), c-Cbl F3 (B) or β-Gal (C, E). The ratio vs c-Cbl F3$^{WT}$ with Src$^{Y527F}$ was averaged from three independent experiments. (mean ± SD; n = 3). * denotes $P < 0.01$ vs c-Cbl F3WT with Src$^{Y527F}$.

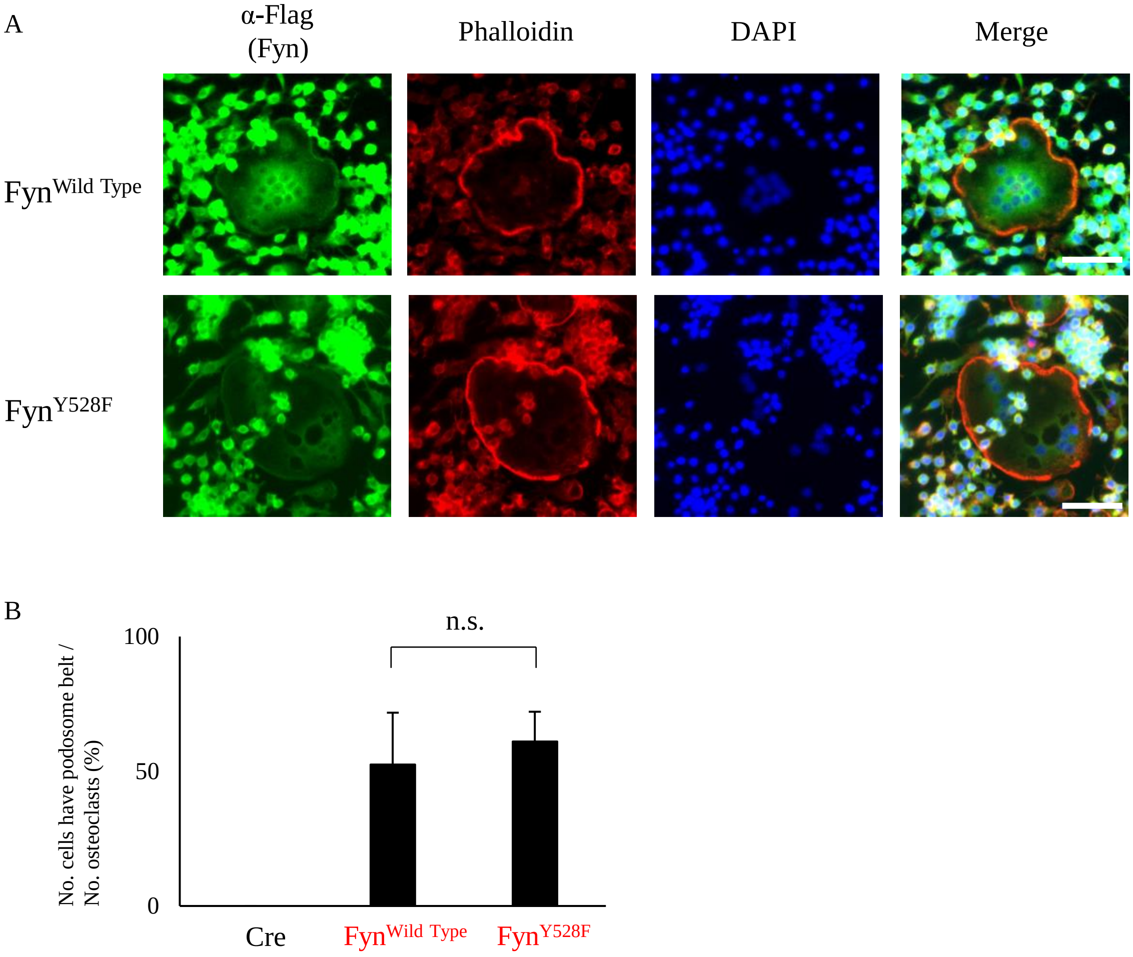


Supplemental Figure 9
(A)Src$^{-/-}$ osteoclasts were infected with Cre adenovirus (M.O.I. 50) and adenoviruses (M.O.I. 50) expressing Fyn$^{wild\ type}$ or Fyn$^{Y528F}$ and cultured for 1 day. Cells were fixed and immunostained with rhodamine phalloidin (red), anti-Flag / anti-mouse alexa fluor488 (green) and DAPI (blue). (B) Osteoclasts and cells having podosome belts were counted to obtain ratio. (mean $\pm$ SD; n = 4). There was no significant difference between Fyn$^{wild\ type}$ and Fyn$^{Y528F}$ (indicated as n.s.). Both Fyn$^{wild\ type}$ and Fyn$^{Y528F}$ were localized around the podosome ring and rescued podosome ring formation.

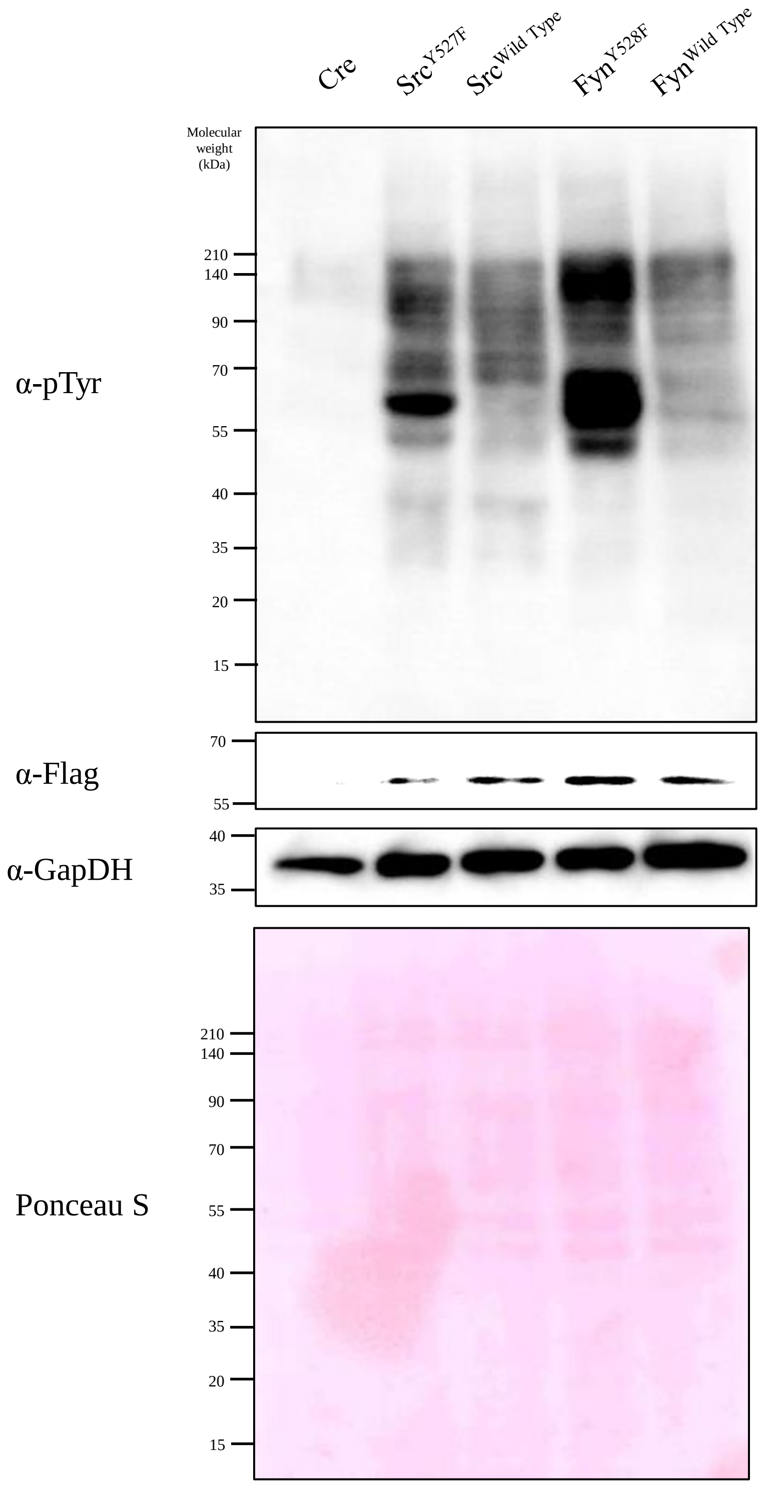


Supplemental Figure 10

$Src^{-/-}$ osteoclasts were infected with Cre adenovirus (M.O.I. 50) and adenoviruses (M.O.I. 50) expressing the indicated wildtype or constitutively activated form of Src family kinases and cultured for 2 days. Cells were lysed and Tyrosine phosphorylation was examined by western blotting analysis.

Similar bands of phospho-tyrosine (pTyr) were detected between $Fyn^{wild\ type}$ and $Fyn^{Y528F}$.
